# Margetuximab with retifanlimab as first-line therapy in HER2+/PD-L1+ unresectable or metastatic gastroesophageal adenocarcinoma: MAHOGANY cohort A

**DOI:** 10.1016/j.esmoop.2022.100563

**Published:** 2022-08-24

**Authors:** D.V.T. Catenacci, Y.-K. Kang, H.H. Yoon, B.Y. Shim, S.T. Kim, D.-Y. Oh, A.I. Spira, S.V. Ulahannan, E.J. Avery, P.M. Boland, J. Chao, H.C. Chung, F. Gardner, S.J. Klempner, K.-W. Lee, S.C. Oh, J. Peguero, M.B. Sonbol, L. Shen, M. Moehler, J. Sun, D. Li, M.K. Rosales, H. Park

**Affiliations:** 1 Department of Medicine, The University of Chicago Medical Centre, Chicago, USA; 2 Department of Oncology, Asan Medical Center, University of Ulsan College of Medicine, Seoul, Republic of Korea; 3 Division of Medical Oncology, Mayo Clinic Comprehensive Cancer Center, Rochester, USA; 4 Medical Oncology, The Catholic University of Korea St. Vincent’s Hospital, Suwon, Republic of Korea; 5 Hematology and Oncology, Samsung Medical Center, Seoul, Republic of Korea; 6 Internal Medicine, Seoul National University Hospital, Cancer Research Institute, Seoul National University College of Medicine, Integrated Major in Innovative Medical Science, Seoul National University Graduate School, Seoul, Republic of Korea; 7 Virginia Cancer Specialists Research Institute, Fairfax, USA; 8 University of Oklahoma Health Sciences Center – Stephenson Cancer Center, Oklahoma City, USA; 9 Division of Hematology and Oncology, Nebraska Hematology-Oncology, Lincoln, USA; 10 Division of Medical Oncology, Rutgers Cancer Institute of New Jersey, New Brunswick, USA; 11 Department of Medical Oncology & Therapeutics Research, City of Hope Comprehensive Cancer Center, Duarte, USA; 12 Department of Medical Oncology, Yonsei Cancer Center, Yonsei University College of Medicine, Seoul, Republic of Korea; 13 Medical Oncology, Florida Cancer Specialists, Cape Coral, USA; 14 Mass General Hospital Cancer Center, Massachusetts General Hospital, Boston, USA; 15 Department of Internal Medicine, Seoul National University College of Medicine, Seoul National University Bundang Hospital, Seongnam; 16 Oncology, Korea University Guro Hospital, Seoul, Republic of Korea; 17 Medical Oncology, Oncology Consultants, Houston, USA; 18 Internal Medicine Department, Mayo Clinic Cancer Center, Phoenix, USA; 19 Department of Gastrointestinal Oncology, Key Laboratory of Carcinogenesis and Translational Research (Ministry of Education/Beijing), Peking University Cancer Hospital & Institute, Beijing, China; 20 Johannes-Gutenberg University, Mainz, Germany; 21 MacroGenics, Inc., Rockville, USA; 22 Department of Medicine, Washington University School of Medicine, St. Louis, USA

**Keywords:** margetuximab, retifanlimab, metastatic gastroesophageal adenocarcinoma, human epidermal growth factor receptor 2, programmed death-ligand 1, first-line therapy

## Abstract

**Background:**

Human epidermal growth factor receptor 2 (HER2)-positive metastatic gastric and gastroesophageal adenocarcinoma (GEA) is globally treated with chemotherapy plus trastuzumab. Novel therapeutic strategies strive to not only optimize efficacy, but also limit toxicities. In MAHOGANY cohort A, margetuximab, an Fc-engineered, anti-HER2 monoclonal antibody (mAb) was combined with retifanlimab, an anti-programmed cell death protein 1 mAb, in the first-line HER2-positive/programmed death-ligand 1 (PD-L1)-positive GEA.

**Patients and methods:**

MAHOGANY cohort A part 1 is a single-arm trial to evaluate margetuximab plus retifanlimab in patients with HER2 immunohistochemistry 3+, PD-L1-positive (combined positive score ≥1%), and non-microsatellite instability-high tumors. Primary objectives for cohort A were safety/tolerability and the confirmed objective response rate (ORR).

**Results:**

As of 3 August 2021, 43 patients were enrolled and received margetuximab/retifanlimab. Nine grade 3 treatment-related adverse events (TRAEs) were reported in eight (18.6%) patients and eight serious TRAEs in seven (16.3%) patients. There were no grade 4/5 TRAEs. Three patients discontinued margetuximab/retifanlimab because of immune-related adverse events. The ORR by independent assessment was 53% [21/40 (95% confidence interval (CI) 36.1-68.5)], with a median duration of response of 10.3 months (95% CI 4.6-not evaluable); disease control rate was 73% [29/40 (95% CI 56.1-85.4)]. The study sponsor discontinued the study in advance of the planned enrollment when it became apparent that the study design would no longer meet the requirements for drug approval because of recent advances in the treatment of GEA.

**Conclusions:**

The chemotherapy-free regimen of combined margetuximab/retifanlimab as first-line treatment in double biomarker-selected patients demonstrated a favorable toxicity profile compared with historical outcomes using chemotherapy plus trastuzumab. The ORR observed in this study compares favorably versus ORR observed with other chemotherapy-free approaches.

## Highlights

• The margetuximab/retifanlimab regimen has a favorable toxicity profile versus historical chemotherapy-based regimens in GEA.
• The margetuximab/retifanlimab regimen as first-line therapy for GEA met the prespecified boundary for antitumor activity.
• The 53% ORR [21/40 (95% CI 36.1-68.5)] in the combined regimen compared favorably with other chemotherapy-free approaches.
• Median duration of response was 10.3 months (95% CI 4.57-not evaluable) and disease control rate was 73% [29/40 (95% CI 56.1-85.4)].
• The study was discontinued for business reasons as chemotherapy-based regimens remain the dominant therapy for GEA.

## Introduction

Novel therapeutic strategies that can not only optimize efficacy but also limit toxicities in newly diagnosed unresectable/metastatic or recurrent, human epidermal growth factor receptor 2 (HER2)-positive gastroesophageal adenocarcinoma (GEA) are needed. HER2 is overexpressed in 15%-25% of patients with GEA.^1^ The Trastuzumab for Gastric Cancer (ToGA) phase III study established the combination with fluoropyrimidine and platinum plus trastuzumab as the standard therapy for HER2-positive GEA.^2^^,^^3^ More recently, other trastuzumab-based chemotherapy-containing combinations have been explored in the phase III studies HELOISE (NCT01450696) and JACOB (NCT01774786).^4^^,^^5^ Across these three studies, objective response rate (ORR) ranged from 47% to 59%, median progression-free survival (PFS) from 5.7 to 7.0 months, and median overall survival (OS) from 12.5 to 14.2 months, with grade ≥3 adverse events (AEs) ranging from 60% to 73% and treatment-related mortality ranging from 2% to 3%.^3^, ^4^, ^5^, ^6^

Margetuximab is an Fc-engineered anti-HER2 monoclonal antibody (mAb) approved in breast cancer^7^ and investigational in GEA, targeting the same epitope as trastuzumab, with increased affinity for both single-nucleotide polymorphisms of the activating Fc receptor (CD16A).^8^, ^9^, ^10^ CD16A is expressed on natural killer cells, natural killer T cells, γδ T cells, dendritic cells, macrophages, and monocytes.^11^ Five amino acid substitutions in the immunoglobulin (Ig) G1 Fc domain of margetuximab lead to higher affinity compared with trastuzumab for both 158V (high-binding) and 158F (low-binding) alleles of the activating FcγRIIIA (CD16A) and diminished binding to inhibitory FcγRIIB (CD32B).^8^^,^^10^ Translational studies suggest that margetuximab may potentially modulate both innate and adaptive immunity, including antigen-specific T- and B-cell responses to HER2.^8^^,^^9^ Programmed death-ligand 1 (PD-L1) positivity by combined positive score (CPS) ≥1 is found in ∼60% of patients with gastric cancer.^12^ Anti-HER2 therapies have been shown to increase PD-L1 expression on tumor cells, pointing to the potential value of adding checkpoint inhibitors to anti-HER2 therapy.^13^

We previously reported findings from a phase Ib/II, open-label, dose-escalation study of margetuximab in combination with pembrolizumab in patients with relapsed/refractory advanced HER2-positive gastroesophageal junction or gastric cancer (CP-MGAH22-05, NCT02689284) that a chemotherapy-free regimen consisting of margetuximab plus pembrolizumab (anti-programmed cell death protein 1 [PD-1] mAb) was well tolerated and induced favorable antitumor activity in patients with previously treated HER2-positive GEA.^14^ In that study, HER2 positivity was defined as immunohistochemistry (IHC) 3+ or IHC2+, and amplified FISH, defined as a HER2 to chromosome enumeration probe 17 ratio ≥2.0 (as per College of American Pathologists/American Society for Clinical Pathology/American Society of Clinical Oncology guidelines). Biomarker analysis revealed an ORR of 44% (11/25) and a disease control rate (DCR) of 72% (18/25) in patients with HER2 IHC3+ and PD-L1 positivity (CPS ≥1, by IHC).^14^ The improved efficacy in tumors with higher expressions of HER2 and PD-L1 was consistent with previous observations evaluating anti-HER2 and anti-PD-1 therapies.^15^, ^16^, ^17^, ^18^, ^19^, ^20^

In a phase II study (NCT02954536) of first-line pembrolizumab in combination with trastuzumab and chemotherapy in HER2-positive GEA, 70% (26/37) of the patients met the primary endpoint of PFS at 6 months.^21^ The ORR was 86% (32/37 patients) and the median duration of response (DOR) was 9.4 months.^21^ However, treatment-related adverse events (TRAEs) of grade 3-4 occurred in 57% (21/37), TRAEs leading to discontinuation occurred in 5% (2/37), and no treatment-related deaths were reported.^21^ In that study, 25 patients were allowed to have one cycle of trastuzumab/pembrolizumab without chemotherapy and restaging after that cycle demonstrated only 8% ORR (2/25).^21^

Retifanlimab (MGA012, INCMGA00012) is an investigational, humanized, hinge-stabilized, IgG4κ anti-PD-1 mAb.^22^ When retifanlimab is used in combination with margetuximab, T cells are sensitized and tumor destruction ensues by enhanced adaptive T-cell-mediated antitumor immunity.^23^

Given this background, we hypothesized that dual blockade targeting HER2 (with margetuximab) and PD-1 (with retifanlimab) would increase antitumor activity by eliciting innate and adaptive immune responses. In order to optimize efficacy and limit toxicity, we enriched for dual biomarker-selected patients (HER2 IHC3+ and PD-L1 positive) in the first-line setting as a chemotherapy-free cohort (cohort A) in the MAHOGANY study (NCT04082364).^23^ This dual selection was mainly based on the 44% ORR found in HER2 IHC3+ and PD-L1-positive patients from the CP-MGAH22-05 study. This article reports the results from MAHOGANY cohort A part 1.

## Patients and methods

### Study design and participants

The MAHOGANY study (NCT04082364) is a phase II/III study in first-line HER2-positive GEA.^23^ Cohort A (Supplementary Figure S1, available at https://doi.org/10.1016/j.esmoop.2022.100563) is a single arm with a Simon two-stage design evaluating efficacy and safety of margetuximab and retifanlimab in patients with HER2 IHC3+ and PD-L1 (CPS ≥1% by IHC with 22C3)-positive tumors, determined by a central laboratory.^24^^,^^25^ In cohort A part 1, efficacy of the combination of margetuximab/retifanlimab is evaluated in ∼40 patients with HER2 IHC3+, PD-L1-positive, nonmicrosatellite instability (MSI)-high tumors. The study was designed to move to cohort A part 2 if the interim analysis, conducted on 40 patients in the responsible evaluable population, passed the prespecified futility border represented by at least 21 (53%) responders (complete response or partial response) per independent review, and the independent data monitoring committee recommended to move to cohort A part 2 to enroll an additional 60 patients. As the interim analysis met futility requirement, five additional patients were enrolled in part 2. However, the sponsor subsequently decided to discontinue enrolling additional patients in cohort A part 2 when new data established the role of chemotherapy-based regimens as the dominant therapy in GEA and that the therapy under study (chemotherapy-free immunotherapy) was not likely to be sufficiently impactful. Trial conduct was in accordance with Good Clinical Practice and Principles in the Declaration of Helsinki. An independent ethics committee approved the protocol at each participating site. All patients provided written informed consent.

### Procedures

Margetuximab 15 mg/kg in combination with retifanlimab 375 mg was administered intravenously every 3 weeks. Efficacy assessments were conducted according to RECIST, version 1.1, every three cycles (±7 days). Survival status was assessed approximately every 3 months for 3 years after study treatment discontinuation. AEs were assessed using the National Cancer Institute Common Terminology Criteria for Adverse Events, version 5.0. An independent data monitoring committee oversaw the ongoing monitoring and interpretation of the safety and efficacy data.

### Objectives

The primary objective for cohort A is the ORR of margetuximab plus retifanlimab in HER2 IHC3+, PD-L1-positive (CPS ≥1), and non-MSI-high patients. Key secondary objectives for cohort A are safety and other efficacy measures including DOR, DCR, PFS, and OS.

### Statistical analysis

The sample size of ∼100 non-MSI-H patients is based on a Simon two-stage design to provide ∼83% power to test ORR of 47% versus 62% at a two-sided α level of 0.05. The null hypothesis H_0_ (47% ORR) would be rejected at a one-sided α level of 0.025 (or equivalently, two-sided 0.05) if the observed ORR from all 100 response-evaluable patients is ≥57%. Cohort A part 1 efficacy and safety were conducted on the first 40 non-MSI-high patients enrolled who were evaluable for response. The safety population includes all patients who receive at least one dose of the study drug. The intention-to-treat population includes all patients who are assigned to treatment. The response-evaluable population includes all patients who received at least one dose of study treatment and had baseline radiographic tumor assessment. The primary analysis of ORR was based on response data by independent assessment. The two-sided 95% exact binomial confidence interval (CI) of ORR and DCR was calculated. The Kaplan–Meier method was applied to estimate DOR, PFS, and OS, respectively. Subgroup analyses of ORR and DOR by PD-L1 CPS status were carried out. After the time of interim analysis, we had 43 patients enrolled in cohort A part 1. The interim analysis was conducted on efficacy data from the protocol-specified response-evaluable population (40 patients) and safety data from the intention-to-treat population (43 patients).

## Results

### Patients

The first patient enrolled in the trial received treatment with margetuximab and retifanlimab combination therapy on 15 October 2019. Among 84 patients screened, 41 failed screening; 30 had biomarker-related issues and 11 had non-biomarker-related issues. Among the 30 screen failures based on biomarker, 21 were HER2 negative only, 3 were PD-L1 negative only, 2 were negative for both biomarkers, and 4 had no biomarker central testing results. As of the 3 August 2021 data cut-off, 43 patients were enrolled and treated; 25 (58%) with gastric cancer and 18 (42%) with GEA, most (84%) with metastatic disease (Table 1). All 43 patients were treated, receiving a median of nine cycles, and a median duration of treatment of 6.6 months (Supplementary Figure S2, available at https://doi.org/10.1016/j.esmoop.2022.100563). Of the 43 treated patients, 20 (46.5%) are continuing to receive the study treatment (Supplementary Figure S2, available at https://doi.org/10.1016/j.esmoop.2022.100563), and 23 (53.5%) discontinued the study treatment. The reasons for discontinuation (*n* = 23) were progressive disease [41.9% (18/43)], AEs [7% (3/43)], and physician decision [4.7% (2/43)]. The median duration of follow-up was 7.6 months among all 43 patients.

**Table 1:** Baseline patient characteristics

Characteristics	ITT population (*N* = 43)
Age, years	
Mean (SD)	64 (11.5)
Median (range)	65 (24-82)
Sex, *n* (%)	
Male	39 (90.7)
Female	4 (9.3)
Race, *n* (%)	
White	20 (46.5)
Asian	19 (44.2)
Black or African American	2 (4.7)
Other/not reported	2 (4.7)
ECOG performance status, *n* (%)	
0	17 (39.5)
1	26 (60.5)
Primary tumor site, *n* (%)	
GC	25 (58.1)
GEJ cancer	18 (41.9)
Extent of the disease at study entry, *n* (%)	
Metastatic	36 (83.7)
Locally advanced	7 (16.3)
Prior anticancer systemic treatment, *n* (%)	
Adjuvant therapy	9 (20.9)
Neoadjuvant therapy	6 (14.0)
Neoadjuvant/adjuvant radiotherapy	9 (20.9)
Prior surgeries with therapeutic intent, *n* (%)	
Total gastrectomy	6 (14.0)
Partial gastrectomy	7 (16.3)
Other	14 (32.6)

Data cut-off 3 August 2021.

ECOG, Eastern Cooperative Oncology Group; GC, gastric cancer; GEJ, gastroesophageal junction; ITT, intention to treat; SD, standard deviation.

### Safety

In the safety population (*n* = 43), the most common TRAEs were fatigue (21%), infusion-related reaction (19%), rash (19%), diarrhea (16%), and pruritus (16%; Table 2). Nine grade 3 TRAEs were reported in 18.6% (8/43) of patients and there were no grade 4 TRAEs (Supplementary Table S1, available at https://doi.org/10.1016/j.esmoop.2022.100563). Eight serious TRAEs were reported in seven (16.3%) patients. Infusion-related reactions considered as AEs of special interest occurred in six (14%) patients. Three (7%) patients discontinued the margetuximab/retifanlimab combination therapy because of the following immune-related AEs: one with grade 3 renal dysfunction, another with grade 3 hepatitis, and the last one with grade 1 diabetic ketoacidosis. Additional immune-related AEs, which did not lead to treatment discontinuation, were grade 1-2 hypothyroidism (*n* = 3) and grade 1-2 pneumonitis (*n* = 2). Dose interruptions of margetuximab resulting from TRAEs occurred in 10 (23%) patients, and in 5 (12%) patients for retifanlimab (Supplementary Table S1, available at https://doi.org/10.1016/j.esmoop.2022.100563). No AEs led to death.

**Table 2:** AEs reported in ≥15% of patients^a^

	Safety population (*N* = 43)			
	TEAEs		TRAEs	
	Any grade, *n* (%)	Grade 3/4, *n* (%)	Any grade, *n* (%)	Grade 3/4, *n* (%)
Any AE	42 (97.7)	18 (41.9)	35 (81.4)	8 (18.6)
Diarrhea	15 (34.9)	2 (4.7)	7 (16.3)	1 (2.3)
Nausea	14 (32.6)	2 (4.7)	4 (9.3)	0 (0)
Anemia	13 (30.2)	4 (9.3)	2 (4.7)	0 (0)
Decreased appetite	11 (25.6)	2 (4.7)	0 (0)	0 (0)
Fatigue	11 (25.6)	1 (2.3)	9 (20.9)	0 (0)
Abdominal pain	10 (23.3)	2 (4.7)	3 (7.0)^b^	0 (0)
Pruritus	10 (23.3)	0 (0)	7 (16.3)	0 (0)
Vomiting	9 (20.9)	1 (2.3)	1 (2.3)	1 (2.3)
Infusion-related reaction	8 (18.6)	0 (0)	8 (18.6)	0 (0)
Rash	8 (18.6)	0 (0)	8 (18.6)	0 (0)
Dyspnea	8 (18.6)	0 (0)	2 (4.7)	0 (0)
Peripheral edema	8 (18.6)	0 (0)	1 (2.3)	0 (0)

Data cut-off 3 August 2021.

AE, adverse event; TEAE, treatment-emergent adverse event; TRAE, treatment-related adverse event.

a Patients are counted only once by preferred term.

b In one patient, abdominal pain was a symptom of an infusion-related reaction.

### Efficacy

**Figure 1:**
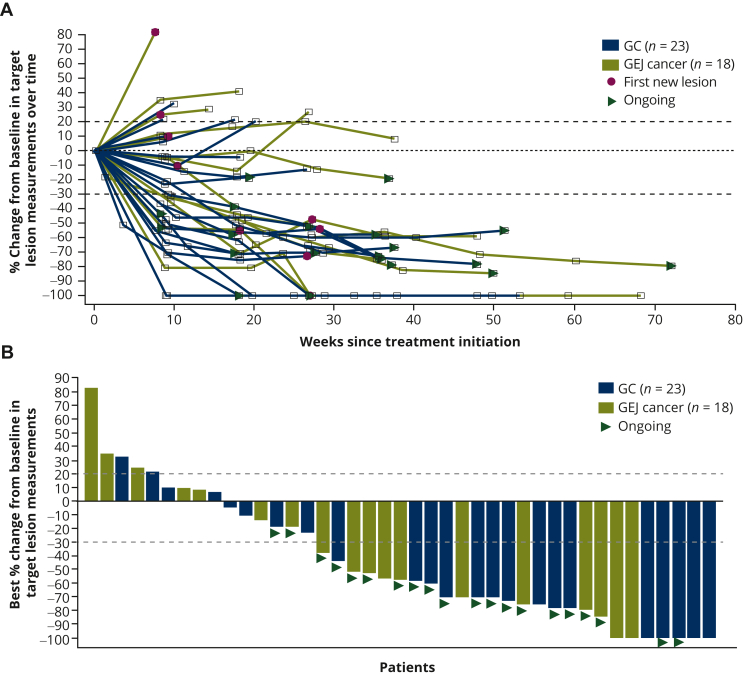
(A) Change in tumor size over time and (B) best change in tumor size by independent assessment (*N* = 41).^a^ Data cut-off 19 July 2021. ^a^ Two patients with GC are not included in these plots as follows: one patient with target lesion not evaluable at postbaseline visit per independent review because of quality of scan imaging and another with only baseline scan assessed by independent review who had clinical progressive disease and discontinued before the first tumor assessment. GC, gastric cancer; GEJ, gastroesophageal junction.

**Figure 2:**
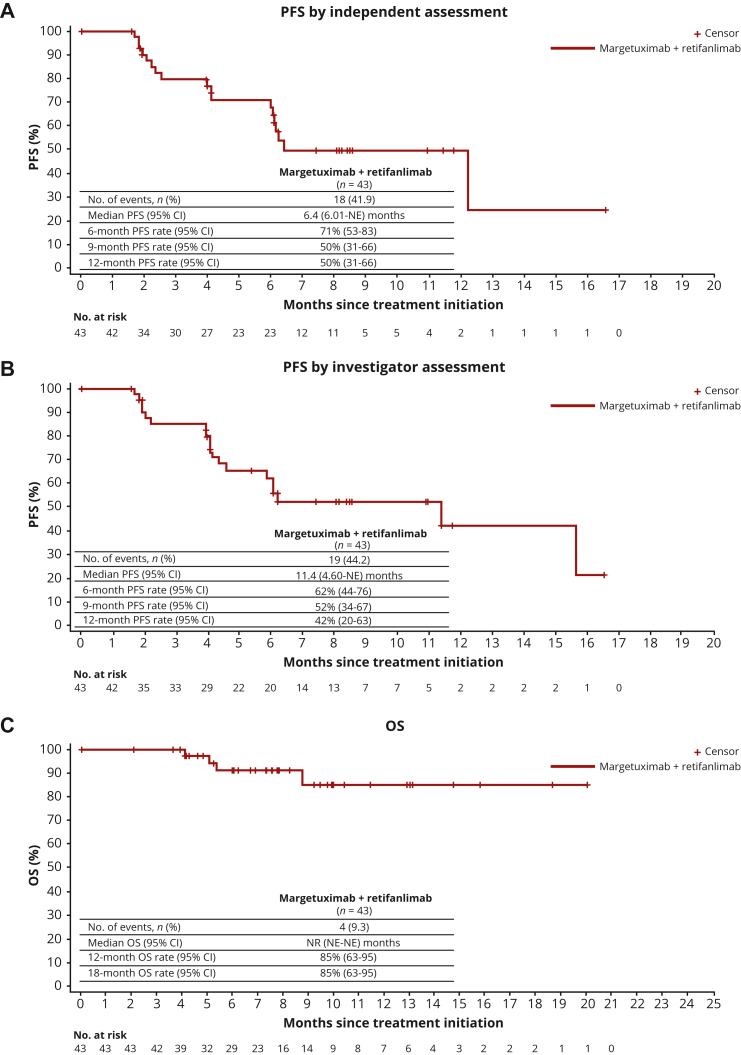
PFS by (A) independent or (B) investigator assessment and (C) OS in the ITT population (*N* = 43). Data cut-off 3 August 2021. CI, confidence interval; ITT, intention to treat; NE, not evaluable; NR, not reached; OS, overall survival; PFS, progression-free survival.

Tumor shrinkage was seen in 32/41 (78%) patients with at least one postbaseline target lesion measurement (Figure 1). The mean best percent change from baseline was −43.95%. Among the first 40 response-evaluable non-MSI-high patients, the ORR by independent assessment was 53% [21/40 (95% CI 36.1-68.5)], with a median DOR of 10.3 months [95% CI 4.6-not evaluable (NE)]; DCR was 73% [29/40 (95% CI 56.1-85.4)] (Table 3). The ORR by investigator assessment was 50% [20/40 (95% CI 33.8-66.2)], with a median DOR of 13.8 months (95% CI 8.8-NE); DCR was 80% [32/40 (95% CI 64.4-90.9)] (Supplementary Table S2, available at https://doi.org/10.1016/j.esmoop.2022.100563). There was high concordance of responders between central independent assessment and investigator assessment (Supplementary Table S3, available at https://doi.org/10.1016/j.esmoop.2022.100563). There were 21 responders per independent assessment versus 20 responders per investigator assessment. There were 18 patients who were classified as responders by both independent and investigator assessments. Analysis of ORR by PD-L1 CPS score showed that ORR was overall similar across the CPS expression subgroups, ranging from 50.0% [12/24 (95% CI 29.1-70.9)] in patients with PD-L1 CPS 1-9 to 56.3% [9/16 (95% CI 29.9-80.2)] in patients with PD-L1 CPS ≥10, with a median DOR of 10.3 months (95% CI 4.3-10.3) or not reached (95% CI 5.3-NE), respectively, per independent assessment (Table 3 and Supplementary Table S2, available at https://doi.org/10.1016/j.esmoop.2022.100563 and Figure S3, available at https://doi.org/10.1016/j.esmoop.2022.100563). There were generally no differences in ORR and DOR by independent assessment between patients with PD-L1 CPS of 1-4 [52.9% (9/17; 95% CI 27.8-77.0); DOR of 10.3 months (95% CI 4.1-10.3)] and the overall population [52.5% (21/40), 95% CI 36.1-68.5; DOR of 10.3 months (95% CI 4.6-NE)]. Most patients who responded had multiple sites of disease (Supplementary Figure S4, available at https://doi.org/10.1016/j.esmoop.2022.100563). Median PFS by independent assessment was 6.4 months (95% CI 6.0-NE) and the 6-month PFS rate was 71% (95% CI 53-83) (Figure 2A). The median PFS by investigator assessment was 11.4 months (95% CI 4.6-NE) and the 6-month PFS rate was 62% (95% CI 44-76) (Figure 2B). The median OS was not reached (Figure 2C).

**Table 3:** Best overall response by independent assessment, overall, and by PD-L1 CPS status

	First 40 response-evaluable patients	
	n	
Best overall response,^a^*n* (%)		
CR		4 (10.0)
PR		17 (42.5)
SD		9 (22.5)
PD		8 (20.0)
NE		2 (5.0)^b^
Objective response (CR + PR), *n* (%); 95% CI	40	21 (52.5); 36.1-68.5
Disease control (CR + PR + SD ≥3 months), *n* (%); 95% CI	40	29 (72.5); 56.1-85.4
Duration of response,^c^ months, median (range); 95% CI	21	10.3 (2.10-14.52); 4.57-NE
Objective response (CR + PR) in PD-L1 CPS 1-4, *n* (%); 95% CI	17	9 (52.9); 27.8-77.0
Disease control (CR + PR + SD ≥3 months) in PD-L1 CPS 1-4, *n* (%); 95% CI	17	11 (64.7); 38.3-85.8
Duration of response^c^ in PD-L1 CPS 1-4, months, median (range); 95% CI	9	10.3 (4.14-10.25); 4.14-10.25
Objective response (CR + PR) in PD-L1 CPS ≥5, *n* (%); 95% CI	23	12 (52.2); 30.6-73.2
Disease control (CR + PR + SD ≥3 months) in PD-L1 CPS ≥5, *n* (%); 95% CI	23	18 (78.3); 56.3-92.5
Duration of response^c^ in PD-L1 CPS ≥5, months, median (range); 95% CI	12	NR (2.10-14.52); 4.57-NE
Objective response (CR + PR) in PD-L1 CPS 1-9, *n* (%); 95% CI	24	12 (50.0); 29.1-70.9
Disease control (CR + PR + SD ≥3 months) in PD-L1 CPS 1-9, *n* (%); 95% CI	24	17 (70.8); 48.9-87.4
Duration of response^c^ in PD-L1 CPS 1-9, months, median (range); 95% CI	12	10.3 (2.33-10.25); 4.30-10.25
Objective response (CR + PR) in PD-L1 CPS ≥10, *n* (%); 95% CI	16	9 (56.3); 29.9-80.2
Disease control (CR + PR + SD ≥3 months) in PD-L1 CPS ≥10, *n* (%); 95% CI	16	12 (75.0); 47.6-92.7
Duration of response^c^ in PD-L1 CPS ≥10, months, median (range); 95% CI	9	NR (2.10-14.52); 5.32-NE

Data cut-off 19 July 2021.

CI, confidence interval; CPS, combined positive score; CR, complete response; GC, gastric cancer; NE, not evaluable; NR, not reached; PD, progressive disease; PD-L1, programmed death-ligand 1; PR, partial response; SD, stable disease.

a CR and PR include only confirmed responses.

b One patient with GC with target lesion not evaluable at post-baseline visit per independent review because of quality of scan imaging and another patient with GC with only baseline scan assessed by independent review (also by investigator) who had clinical progressive disease and discontinued before the first tumor assessment.

c Calculated only for patients with objective response of CR or PR.

## Discussion

In cohort A part 1 of MAHOGANY, patients with HER2 IHC3+ and PD-L1-positive GEA received the chemotherapy-free regimen of margetuximab and retifanlimab, and most patients (78%) had tumor shrinkage at first scan. The number of confirmed responders [21/40 (53%); median DOR of 10.3 months] was determined by independent assessment. Concordance of responders assessed by independent review or by the investigator was high. Analysis of ORR by PD-L1 CPS score showed that this regimen provides a good response regardless of PD-L1 CPS score (ORR ranging from 50% to 56%), with the highest ORR and median DOR in patients with PD-L1 CPS ≥10 (ORR of 56%; median DOR was not reached).

Antitumor activity of the MAHOGANY cohort A chemotherapy-free combination was comparable to historical data of trastuzumab plus chemotherapy from the ToGA study (*n* = 294; ORR of 47%; median DOR of 6.9 months),^3^ the HELOISE study (*n* = 124; ORR of 59%),^4^ and the JACOB study (*n* = 392; ORR of 48%; median DOR of 8.4 months),^5^ as well as initial data from the control arm (placebo + trastuzumab + chemotherapy) of the KEYNOTE-811 (NCT03615326) study (*n* = 131; ORR of 52%; median DOR of 9.5 months).^26^ Importantly, the ORR of 53% observed in MAHOGANY cohort A part 1 in first-line HER2 IHC3+/PD-L1-positive GEA compares favorably versus ORR observed with other chemotherapy-free approaches, such as pembrolizumab monotherapy demonstrating an ORR of 15% in first-line HER2-negative/PD-L1-positive (CPS ≥1) and 25% in CPS ≥10 GEA (KEYNOTE-062, NCT02494583),^27^ as well as an ORR of 16% in second-line PD-L1-positive (CPS ≥1) GEA (KEYNOTE-061, NCT02370498).^20^ Moreover, among CPS ≥1 tumors in first-line KEYNOTE-062 treated with pembrolizumab monotherapy, the DCR was only 42% and only slightly higher DCR of 50% in CPS ≥10.^27^ Further, in the CPS ≥1 population, the median PFS was only 2.0 months with an early and high incidence of death compared with standard chemotherapy, despite this biomarker selection.^27^ Thus, the results of MAHOGANY cohort A far exceed these outcomes of pembrolizumab monotherapy, likely owing to the dual biomarker selection imposed for eligibility, and therefore mitigating concerns of inadequate efficacy with a chemotherapy-free approach in these patients.

Recent studies suggest that additional anti-PD-1 agents increase efficacy of chemotherapy in HER2-negative GEA, particularly in tumors with higher PD-L1 expression. Pembrolizumab, in combination with chemotherapy, received United States Food and Drug Administration (FDA) approval irrespective of PD-L1 score in the first-line setting, based on KEYNOTE-590 (NCT03189719).^28^^,^^29^ The European Medicines Agency (EMA) approved pembrolizumab in combination with chemotherapy for the first-line HER2-negative advanced or metastatic esophageal and gastroesophageal junction cancers with PD-L1 CPS ≥10.^30^ According to the National Comprehensive Cancer Network (NCCN) guidelines, in HER2-negative esophageal and gastroesophageal junction cancers, pembrolizumab in combination with chemotherapy is recommended as category 2B in patients with PD-L1 CPS <10, and as category 1 in patients with PD-L1 CPS ≥10.^31^ The anti-PD-1 mAb nivolumab in combination with chemotherapy received FDA approval for the first-line treatment of patients with advanced or metastatic GEA regardless of PD-L1 expression, based on CHECKMATE-649 (NCT02872116).^32^^,^^33^ The EMA approved nivolumab in combination with chemotherapy for the first-line treatment of adult patients with HER2-negative advanced or metastatic GEA with PD-L1 CPS ≥5.^34^ According to the NCCN guidelines, in HER2-negative GEA nivolumab in combination with chemotherapy is recommended as category 1 in patients with PD-L1 CPS ≥5 and as category 2B in patients with PD-L1 CPS <5.^31^^,^^35^ In the setting of both monotherapy and combination with chemotherapy, in HER2-negative GEA, the thresholds used to evaluate the efficacy of anti-PD-1 agents by PD-L1 expression were ‘CPS ≥1’ and ‘CPS ≥10’ for pembrolizumab (KEYNOTE-061, KEYNOTE-062, and KEYNOTE-590),^20^^,^^27^^,^^29^ and ‘CPS ≥5’ for nivolumab (CHECKMATE 649).^33^

In HER2-positive GEA, increased tumor responses with anti-PD-1 antibody and HER2 blockade were reported. Pembrolizumab, in combination with trastuzumab and chemotherapy, received an accelerated approval from the FDA for the first-line treatment of patients with advanced HER2-positive (HER2 IHC3+ or HER2 IHC2+/FISH positive) GEA, regardless of PD-L1 expression based on KEYNOTE-811, where the ORR was 74% in the pembrolizumab arm versus 52% in the placebo arm (median DOR was 10.6 months versus 9.5 months).^26^^,^^28^ The incidence of patients with tumors PD-L1 CPS ≥1 was 86%. According to the NCCN guidelines, in HER2-positive GEA, pembrolizumab in combination with trastuzumab and chemotherapy is recommended as category 2A regardless of PD-L1 expression.^31^^,^^35^ Other studies are evaluating combination of anti-HER2 plus anti-PD-1 strategies in combination with chemotherapy. Preliminary results from the phase II study (NCT03929666) of the anti-HER2 bispecific (binding ECD4 and ECD2) mAb zanidatamab plus chemotherapy as first-line treatment in 28 patients with advanced HER2-positive GEA showed an ORR of 75% and a median DOR of 16.4 months.^36^ A phase Ib/II study (NCT04276493) is ongoing, investigating zanidatamab plus chemotherapy with the investigational anti-PD-1 agent tislelizumab as first-line treatment for patients with advanced HER2-positive GEA. In addition, recent data from triple combination with anti-PD-1, trastuzumab, and chemotherapy have shown ORRs of 77%^37^ and 86%^21^ for pembrolizumab, and 61%^38^ for avelumab. These improvements in ORR are encouraging, and whether this translates into longer-term benefit including PFS and OS is awaited with longer follow-up. Evaluation by PD-L1 status at the pertinent cut-offs (CPS ≥1, ≥5, ≥10) is also awaited to determine which scenarios that immunotherapy provides the most therapeutic value. Moreover, the toxicity profiles of these chemotherapy-containing regimens^6^ must be considered to determine optimal treatment strategies that can be personalized for each patient.

The safety findings on 43 patients enrolled in MAHOGANY cohort A and treated with margetuximab plus retifanlimab suggest that this chemotherapy-free combination regimen was well tolerated. Treatment-emergent AEs of grade 3-4 occurred in 41.9% (18/43) of patients, TRAEs of grade 3-4 occurred in 18.6% (8/43) of patients, 7.0% (3/43) of patients discontinued study treatment due to TRAEs (immune-related renal dysfunction, immune-related hepatitis, and diabetic ketoacidosis), and no AEs led to death. MAHOGANY cohort A safety data compare favorably to ToGA,^3^ the initial results from KEYNOTE-811,^26^ and the preliminary results from the zanidatamab plus chemotherapy phase II study (NCT03929666).^36^ According to recent studies on triple combination with anti-PD-1, trastuzumab, and chemotherapy, TRAEs of grade 3-5 were reported in 57%^21^-81%^37^ of patients, treatment-related mortality in 0%^21^-3%^26^ of patients, and TRAEs leading to discontinuation in 5%^21^-24%^26^ of patients. Despite limitations of cross-study comparisons, it seems clear that there are clinically relevant toxicity differences between regimens containing chemotherapy versus those without chemotherapy, notably AEs of grade 3-4 and AEs leading to death (grade 5) or treatment discontinuation. In this study, there were no grade 4 TRAEs or grade 5 AEs.

In summary, the chemotherapy-free regimen combining margetuximab and retifanlimab as first-line treatment in biomarker-selected patients (HER2 IHC3+, PD-L1 positive) met the prespecified boundary for antitumor activity and demonstrated a favorable toxicity profile compared with historical outcomes using chemotherapy plus trastuzumab.^6^ Moreover, initiating chemotherapy only if patients experienced disease progression did not appear to affect OS negatively; thus, delaying cytotoxic therapy allowed for patients to suspend experiencing chemotherapy-related toxicity, such as cumulative neuropathy. In a subset of patients with profound and durable response, this strategy may spare patients from chemotherapy exposure altogether, thus limiting overtreatment in this biomarker-selected population. The sponsor decided to discontinue enrollment in cohort A part 2 for business reasons, including that chemotherapy continues to make significant contributions in battling GEA, while chemotherapy-free immunotherapy of this type is less effective than hoped.
