# Supplementary material for: Margetuximab with retifanlimab as first-line therapy in HER2+/PD-L1+ unresectable or metastatic gastroesophageal adenocarcinoma: MAHOGANY cohort A
Source: ESMO Open. 2022 Aug 24;7(5):100563. doi: 10.1016/j.esmoop.2022.100563 (PMC9588876; doi:10.1016/j.esmoop.2022.100563)
Supplement: Supplementary Material [file mmc1.docx]

**Supplementary material**

**Table S1. Safety summary**

|  | **Safety population (*N* = 43)** | |
| --- | --- | --- |
|  | **TEAE, *n* (%)** | **TRAE, *n* (%)** |
| Any AE | 42 (97.7) | 35 (81.4) |
| Any grade 3/4 AE | 18 (41.9) | 8 (18.6) |
| Any SAE | 14 (32.6) | 7 (16.3) |
| Any AE resulting in death | 0 | 0 |
| AEs leading to margetuximab discontinuation | 3 (7.0) | 3 (7.0) |
| AEs leading to retifanlimab discontinuation | 3 (7.0) | 3 (7.0) |
| AEs leading to margetuximab interruption | 12 (27.9) | 10 (23.3) |
| AEs leading to retifanlimab interruption | 8 (18.6) | 5 (11.6) |

Data cutoff August 3, 2021.

AE, adverse event; SAE, serious adverse event; TEAE, treatment-emergent adverse event; TRAE, treatment-related adverse event.

**Table S2. Best overall response by investigator assessment, overall, and by PD-L1 CPS status**

|  | **First 40 response-evaluable patients** | |
| --- | --- | --- |
|  | ***n*** |  |
| Best overall response,^a^ *n* (%) |  |  |
| CR |  | 4 (10.0) |
| PR |  | 16 (40.0) |
| SD |  | 13 (32.5) |
| PD |  | 6 (15.0) |
| NE |  | 1 (2.5)^b^ |
| Objective response (CR + PR), *n* (%) [95% CI] | 40 | 20 (50.0) [33.8-66.2] |
| Disease control (CR + PR + SD ≥3 months), *n* (%) [95% CI] | 40 | 32 (80.0) [64.4-90.9] |
| Median duration of response,^c^ (min, max) [95% CI], months | 20 | 13.8 (2.10, 14.52) [8.80-NE] |
|  |  |  |
| Objective response (CR + PR) in PD-L1 CPS 1-4, *n* (%) [95% CI] | 17 | 8 (47.1) [23.0-72.2] |
| Disease control (CR + PR + SD ≥3 months) in PD-L1 CPS 1-4, *n* (%) [95% CI] | 17 | 13 (76.5) [50.1-93.2] |
| Median duration of response^c^ in PD-L1 CPS 1-4, (min, max) [95% CI], months | 8 | 13.8 (4.14, 13.83) [4.14-13.83] |
|  |  |  |
| Objective response (CR + PR) in PD-L1 CPS ≥5, *n* (%) [95% CI] | 23 | 12 (52.2) [(30.6-73.2)] |
| Disease control (CR + PR + SD ≥3 months) in PD-L1 CPS ≥5, *n* (%) [95% CI] | 23 | 19 (82.6) [61.2-95.0] |
| Median duration of response^c^ in PD-L1 CPS ≥5, (min, max) [95% CI], months | 12 | NR (2.10, 14.52) [4.14-NE] |
|  |  |  |
| Objective response (CR + PR) in PD-L1 CPS 1-9, *n* (%) [95% CI] | 24 | 11 (45.8) [25.6-67.2] |
| Disease control (CR + PR + SD ≥ 3 months) in PD-L1 CPS 1-9, *n* (%) [95% CI] | 24 | 19 (79.2) [57.8-92.9] |
| Median duration of response^c^ in PD-L1 CPS 1-9, (min, max) [95% CI], months | 11 | 13.8 (2.33, 13.83) [4.14-13.83] |
|  |  |  |
| Objective response (CR + PR) in PD-L1 CPS ≥10, *n* (%) [95% CI] | 16 | 9 (56.3) [29.9-80.2] |
| Disease control (CR + PR + SD ≥3 months) in PD-L1 CPS ≥10, *n* (%) [95% CI] | 16 | 13 (81.3) [54.4-96.0] |
| Median duration of response^c^ in PD-L1 CPS ≥10, (min, max) [95% CI], months | 9 | NR (2.10, 14.52) [NE-NE] |

Data cutoff July 19, 2021.

CI, confidence interval; CPS, combined positive score; CR, complete response; max, maximum; min, minimum; NE, not evaluable; NR, not reached; PD, progressive disease; PD-L1, programmed death-ligand 1; PR, partial response; SD, stable disease.

^a^CR and PR include only confirmed responses.

^b^One patient with gastric cancer with only baseline scan assessed by investigator (also by independent review) who had clinical progressive disease and discontinued before the first tumor assessment.

^c^Calculated only for patients with objective response of CR or PR.

**Table S3.** **Cross-classification of independently assessed BOR and investigator-assessed BOR (*N* = 40)**

|  | **Independently assessed BOR** | | | | |  |
| --- | --- | --- | --- | --- | --- | --- |
| **Investigator-assessed BOR** | **CR** | **PR** | **SD** | **PD** | **NE** | **Total** |
| CR | 2 | 2 | 0 | 0 | 0 | 4 |
| PR | 1 | 13 | 2 | 0 | 0 | 16 |
| SD | 1 | 2 | 6 | 3 | 1 | 13 |
| PD | 0 | 0 | 1 | 5 | 0 | 6 |
| NE | 0 | 0 | 0 | 0 | 1 | 1 |
| Total | 4 | 17 | 9 | 8 | 2 | 40 |

Data cutoff July 19, 2021.

BOR, best overall response; CR, complete response; NE, not evaluable; PD, progressive disease; PR, partial response; SD, stable disease.

# Figure S1. MAHOGANY Cohort A: A nonrandomized, single-arm, open-label study testing a chemotherapy-free regimen.

#
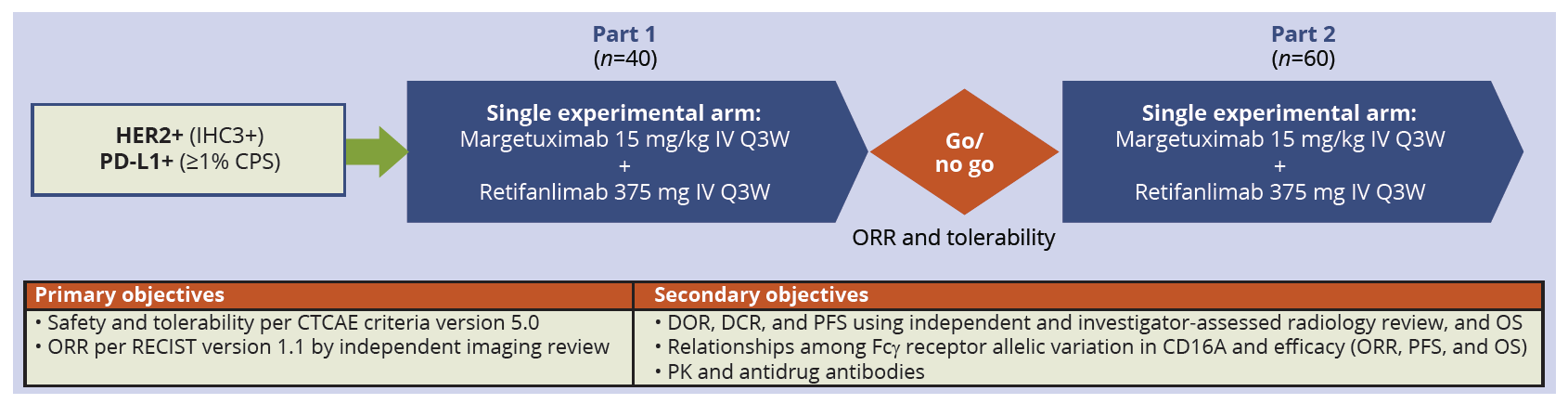


# CPS, combined positive score; CTCAE, Common Terminology Criteria for Adverse Events; DCR, disease control rate; DOR, duration of response; HER2+, human epidermal growth factor receptor 2 positive; IHC, immunohistochemistry; IV, intravenous; ORR, objective response rate; OS, overall survival; PD-L1, programmed death-ligand 1; PFS, progression-free survival; PK, pharmacokinetic; Q3W, every 3 weeks; RECIST, Response Evaluation Criteria in Solid Tumors.

**Figure S2. Duration of treatment by primary tumor site in the ITT population (*N*=43)**


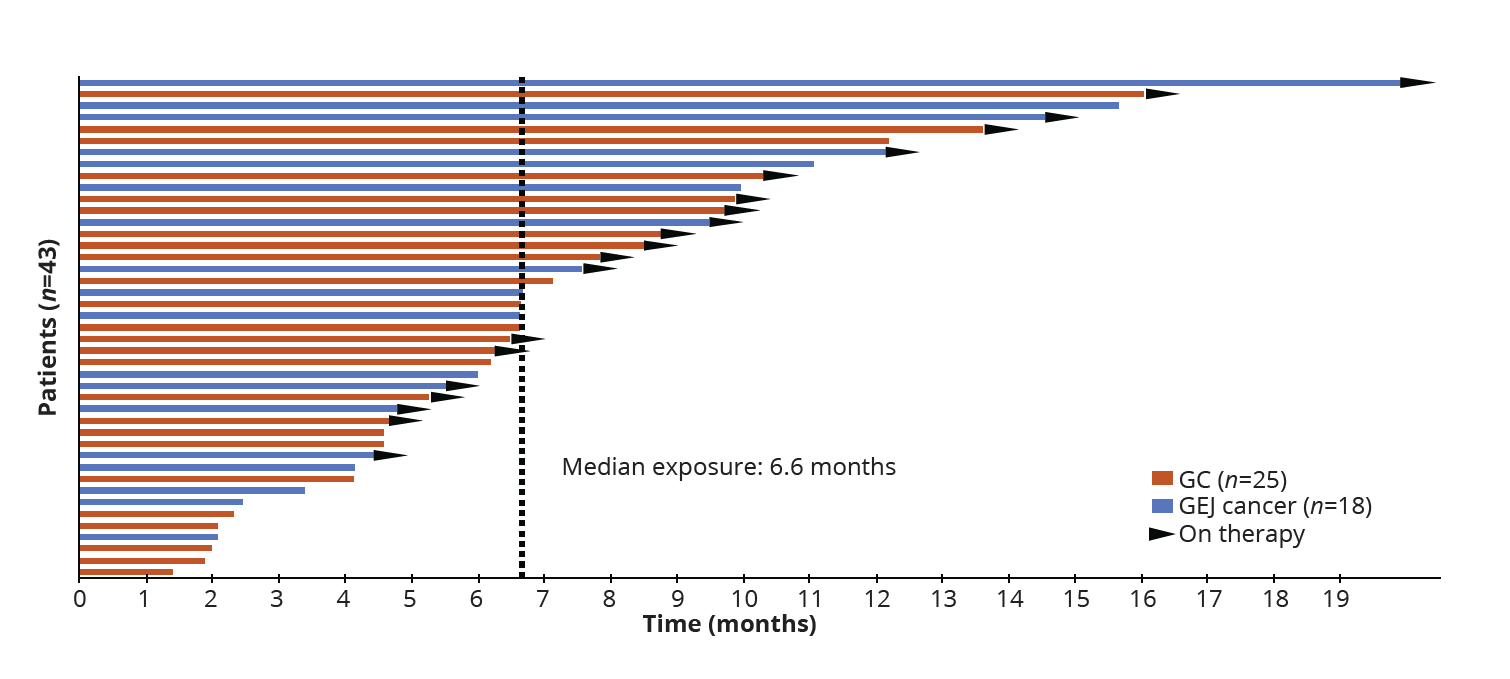


Data cutoff August 3, 2021.
GC, gastric cancer; GEJ, gastroesophageal junction; ITT, intention-to-treat.

**Figure S3. DOR by independent assessment and by investigator assessment in the responders, by PD-L1 CPS status.**


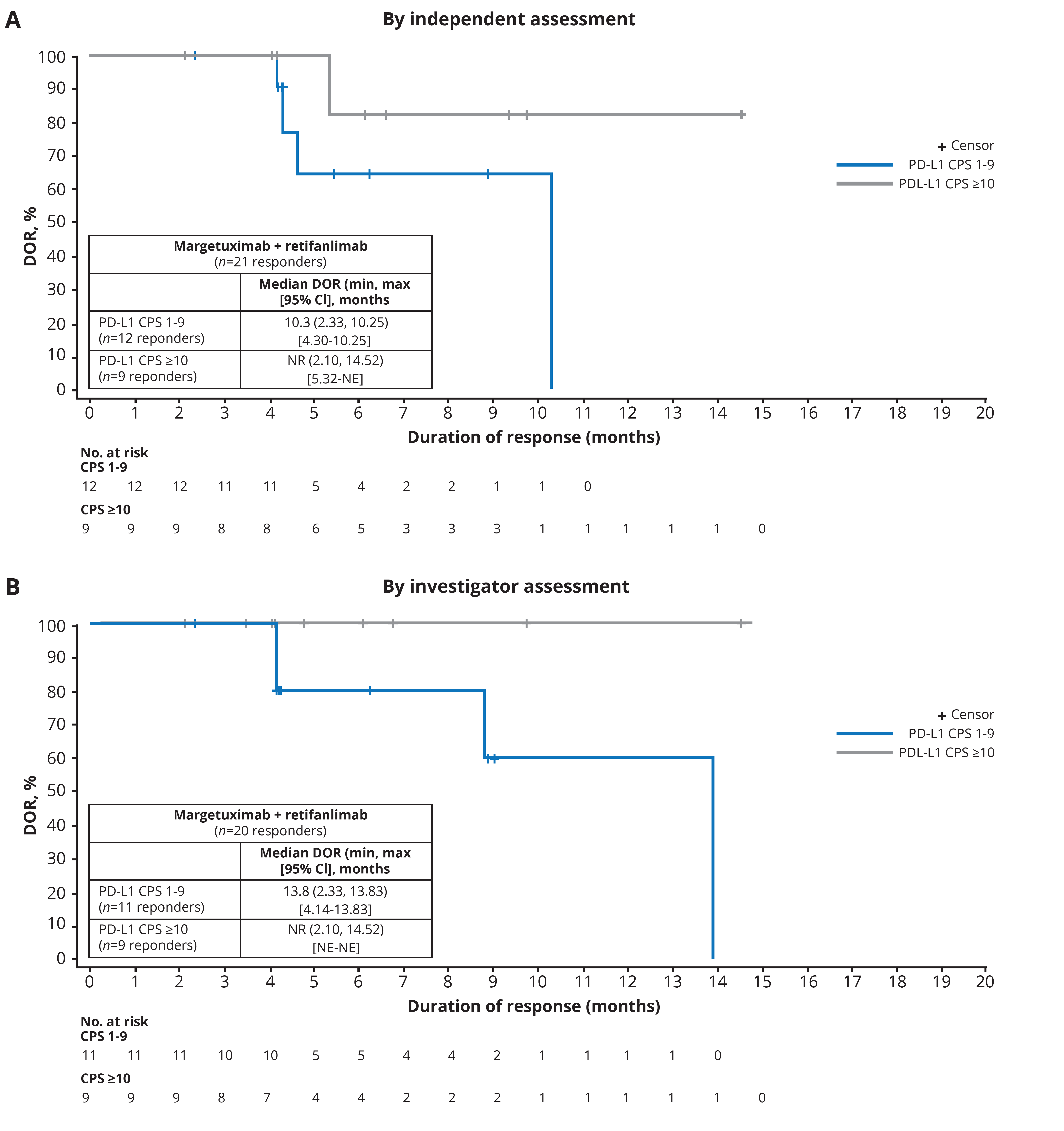


CI, confidence interval; CPS, combined positive score; DOR, duration of response; max, maximum; min, minimum; PD-L1, programmed death-ligand 1.

**Figure S4. Radiographic scans of 2 patients who achieved PRs after treatment with margetuximab + retifanlimab.**

**
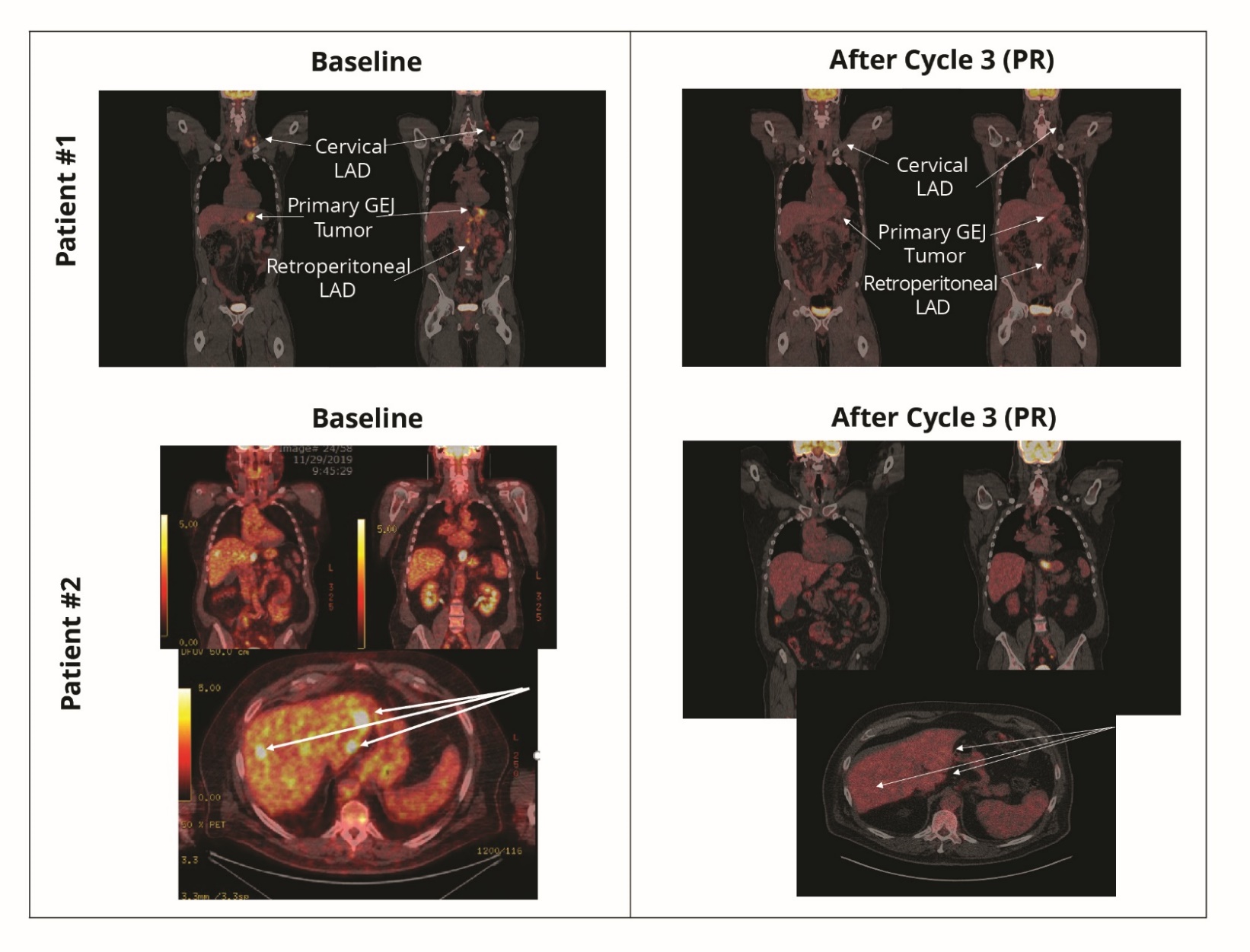
**

GEJ, gastroesophageal junction; LAD, lymphadenopathy; PR, partial response.
